# Collection of Partition Coefficients in Hexadecyltrimethylammonium Bromide, Sodium Cholate, and Lithium Perfluorooctanesulfonate Micellar Solutions: Experimental Determination and Computational Predictions

**DOI:** 10.3390/molecules28155729

**Published:** 2023-07-28

**Authors:** Leila Saranjam, Miroslava Nedyalkova, Elisabet Fuguet, Vasil Simeonov, Francesc Mas, Sergio Madurga

**Affiliations:** 1 Department of Material Science and Physical Chemistry, Research Institute of Theoretical and Computational Chemistry (IQTCUB), University of Barcelona, C/Martí i Franquès 1, 08028 Barcelona, Spain; lsaransa19@alumnes.ub.edu (L.S.); fmas@ub.edu (F.M.); 2 Faculty of Chemistry and Pharmacy, University of Sofia “St. Kl. Ohridski”, 1 James Bourchier Blvd., 1164 Sofia, Bulgaria; ahvs@chem.uni-sofia.bg; 3 Department of Chemical Engineering and Analytical Chemistry, Institute of Biomedicine (IBUB), University of Barcelona, C/Martí i Franquès 1, 08028 Barcelona, Spain; elifuguetj@ub.edu; 4 Serra Húnter Programme, Generalitat de Catalunya, 08017 Barcelona, Spain

**Keywords:** partition coefficient, micelle, hexadecyltrimethylammonium bromide (HTAB), sodium cholate (SC), lithium perfluorooctanesulfonate (LPFOS), SVM, DFT, k-means clustering

## Abstract

This study focuses on determining the partition coefficients (logP) of a diverse set of 63 molecules in three distinct micellar systems: hexadecyltrimethylammonium bromide (HTAB), sodium cholate (SC), and lithium perfluorooctanesulfonate (LPFOS). The experimental log *p* values were obtained through micellar electrokinetic chromatography (MEKC) experiments, conducted under controlled pH conditions. Then, Quantum Mechanics (QM) and machine learning approaches are proposed for the prediction of the partition coefficients in these three micellar systems. In the applied QM approach, the experimentally obtained partition coefficients were correlated with the calculated values for the case of the 15 solvent mixtures. Using Density Function Theory (DFT) with the B3LYP functional, we calculated the solvation free energies of 63 molecules in these 16 solvents. The combined data from the experimental partition coefficients in the three micellar formulations showed that the 1-propanol/water combination demonstrated the best agreement with the experimental partition coefficients for the SC and HTAB micelles. Moreover, we employed the SVM approach and k-means clustering based on the generation of the chemical descriptor space. The analysis revealed distinct partitioning patterns associated with specific characteristic features within each identified class. These results indicate the utility of the combined techniques when we want an efficient and quicker model for predicting partition coefficients in diverse micelles.

## 1. Introduction

The partition coefficient (logP) is a significant physicochemical parameter used in various fields such as drug and pharmaceutical product design, substance toxicology, and environmental fate modeling of organic compounds [1]. It measures the solute’s solubility in two immiscible solvents, providing valuable insights into solute distribution. In drug delivery systems, the partition coefficient is crucial in determining the system’s ability to distribute molecules between the aqueous phase and micelles [2]. Regular micelles consist of a polar head and a nonpolar tail, enabling the dissolution of both polar and nonpolar molecules. Hydrophilic solutes preferentially interact with the polar, hydrophilic surface of the micelle, while hydrophobic solutes tend to accumulate in the nonpolar, hydrophobic core of the micelle [3,4]. Understanding the partitioning behavior within micelles can contribute to enhancing the efficacy and safety of drug delivery systems. Moreover, this knowledge can be leveraged to optimize the design and performance of such systems, leading to improved therapeutic outcomes [5,6]. When hydrophobic compounds are introduced into micellar solutions, they have a higher tendency to associate with the micelles because the hydrophobic regions of the micelles (the inner hydrophobic tails) can provide a more favorable environment for the hydrophobic molecules. As a result, more hydrophobic molecules have higher values of partition coefficients in micellar systems. In this context, the use of pluronic micelles for delivering hydrophobic drugs presents an interesting and alternative approach [7,8].

Various experimental methods can be used to estimate the micelle–water partition coefficient, such as solubility analysis, micellar-enhanced ultrafiltration, micellar liquid chromatography [9,10], and cloud-point extraction [11]. In this study, the micelle–water partition coefficients were estimated from the retention times of micellar electrokinetic chromatography (MEKC) experiments [12,13].

The MEKC technique is widely employed for the separation and identification of components within a mixture. This technique utilizes as a pseudo-stationary phase a surfactant above its critical micellar concentration (CMC) to facilitate the formation of micelles in an aqueous solution. By applying an electric field, the components within the mixture are partitioned between the aqueous and the micellar phases, leading to their separation. MEKC separations present high resolution and efficiency in the analysis of both neutral and charged compounds. Moreover, the separations can be easily optimized just by changing the nature of the surfactant [14,15]. This technique has been used for the determination of partition coefficients in micelles in many different fields [16,17,18,19,20].

From a computational point of view, molecular dynamics (MD) simulations could provide valuable insights into the transfer of solutes between different phases, such as from the aqueous phase to the micellar phase [4]. These simulations allow free energy profiles to be obtained, which quantitatively describe the energetic changes associated with solute transfer. In the context of drug delivery systems, these profiles help in understanding the distribution of drugs within micelles and optimize their design. However, it is important to note that molecular dynamics simulations can be computationally costly because of the need for long converged trajectories.

MD simulations combined with the COSMOmic method have been shown to be a promising alternative [5,21,22] to experimental methods for predicting the partition coefficient in micellar systems. Previous studies have demonstrated good correlation between predicted and experimental data for a variety of micelles [23], including sodium dodecyl sulfate (SDS), hexadecyltrimethylammonium bromide (HTAB, also known as Cetyltrimethylammonium Bromide, CTAB), sodium cholate (SC), lithium perfluorooctanesulfonate (LPFOS), C_12_E_10_, Brij35, Triton X-114, and Triton X-100.

Recently, a study on mixed micelles formed by sodium laureth sulfate (SLES) and fatty acids, using molecular dynamics simulations, shows that the micelle–water partition coefficients of neutral and charged fatty acids could be calculated using the COSMOmic and the MD approach [24]. Based on the potential of mean force (PMF) calculations performed using umbrella sampling (US), the study shows that the partition coefficients for neutral solutes can be accurately calculated using both the COSMOmic and additive CGenFF US/PMF approaches, while the Drude polarizable force field is needed to accurately calculate the experimental partition coefficient of the charged solute. There are other examples of MD simulations with US and COSMOmic [25], demonstrating the utility of these methods for predicting partition equilibria in micellar systems.

Moreover, the fragmental constant method (FCA) has also been applied to determine partition coefficients. The FCA model defines a micelle–water partition coefficient as the sum of the partition coefficients of the component’s atomic/molecular fragments, determined by fragmental constant values [26]. Fragmental techniques are ineffective at estimating the parameters of other solvents and are only appropriate for a narrow range of solvents (typically octanol/water).

Alternatively, other ways of categorizing logP predictors use parametric models, which employ methods such as least squares estimation or multiple linear regression to fit the parameters governing the relative contributions of different input features. Machine-learning-based methods, including Support Vector Machines (SVM) [27,28], Neural Networks (NNs) [29], and Graph Convolutional Networks (GCN) [30], have also been utilized for logP prediction. In a recent study by Dickson et al. [31], various methods for predicting logP values in a dataset of small molecules were examined. The study focused on transforming atomic properties, such as radius and partial charge, which are commonly employed as force field parameters in classical molecular dynamics simulations. These attributes were converted into index-invariant molecular features using a recently developed technique known as geometric scattering for graphs (GSG) [31]. The results obtained from this investigation demonstrate that the most accurate predictions were achieved using atomic attributes generated with the CHARMM generalized force field and 2D molecular structures. This highlights the significance of employing appropriate molecular representations and force field parameters for accurate logP prediction.

Here, the focus is on the use of density functional theory (DFT) calculations and SVM calculations for predicting the partition coefficients of compounds in micellar systems. DFT calculations are a Quantum Mechanics (QM) computational method that can be used to predict the partition coefficients of compounds in micellar systems. They are faster and less computationally demanding than molecular dynamics simulations, making them an attractive alternative for predicting the properties of drugs in micellar systems. By calculating the energy changes associated with transferring a compound from the aqueous phase to a solvent phase that resembles the behavior of the micellar phase, DFT calculations can provide an estimate of the compound’s partition coefficient in a micellar system. This makes DFT calculations a valuable tool for drug delivery design and optimization. The first step is to use DFT calculations to identify the combination of solvents that can best predict the experimental partition coefficients of compounds in a specific micellar system. This study aims to apply the DFT calculation approach to predict the partition coefficients of 63 compounds in HTAB, SC, and LPFOS micellar solutions (Table 1). The study will compare the predicted partition coefficients with experimental data to assess the accuracy of the DFT approach. The prediction of 15 solvent–water partition coefficients is achieved by applying DFT with the B3LYP method [32] with a 6-31++G** basis set. The solvation model based on the density (SMD) is applied to evaluate the free energy of solvation [33,34]. This model divides the solvation free energy into two main contributions—the bulk electrostatic contribution and the cavity dispersion contribution—and it can be applied to any charged or uncharged solute in any type of solvent as a universal solvation model. Using this approach, correlations with micellar partition coefficients in SC, HTAB, and LPFOS micellar systems are performed.

Finally, SVM calculations are performed using the experimental values obtained from the three micellar systems. SVM calculations involve the application of a supervised machine learning algorithm widely utilized in pattern recognition and regression tasks. SVM-based models can capture complex relationships between molecular descriptors and partition coefficients, thereby enabling the prediction of partition coefficients for a diverse range of compounds. The utilization of SVM calculations in partition coefficient prediction offers several advantages. Firstly, it enables the rapid and cost-effective screening of large compound libraries, facilitating the identification of promising candidates for drug development or the assessment of environmental impact. Additionally, SVM models can accommodate a wide range of chemical structures and properties, making them applicable to various classes of compounds. Moreover, SVM-based models can incorporate both structural and physicochemical descriptors, providing a comprehensive representation of the molecular characteristics that influence partitioning behavior. This facilitates the exploration of structure–activity relationships and the identification of key features contributing to partition coefficients, thereby assisting in the design and optimization of compounds with desired properties. Furthermore, the predictive accuracy of SVM models can be continuously improved by incorporating more diverse and high-quality data, as well as by optimizing the selection and combination of molecular descriptors. As a result of this iterative process, the models are refined and their reliability and robustness are enhanced.

## 2. Results and Discussion

The experimental values of logP obtained from the SC, LPFOS, and HTAB micelles were analyzed and used to parametrize the computational methodology applied for each type of micelle. Initially, the logP values were estimated based on simple DFT calculations of the molecules in different solvents. Subsequently, SVM predictions were made after conducting a study on the most relevant descriptors using k-means clustering and PCA.

### 2.1. Experimental logP Values of SC, HTAB, and LPFOS Micelles

The experimental partition coefficients (logP values) in three different types of micelles, namely SC, LPFOS, and HTAB, are presented in Table 2. These logP values were determined by measuring the retention factors of the compounds in 80 mM SC micelles in 20 mM phosphate buffer, 40 mM LPFOS micelles in 20 mM phosphate buffer, and 20 mM HTAB micelles in 20 mM phosphate buffer at pH 7 and 25 °C. The logP values of 63 compounds, representing a diverse set of compounds including benzene derivatives, nitrogen-containing heterocycles, pesticides, hormones, and pharmaceutical compounds, are displayed in Table 2. The selection of compounds was performed according to a previous study [13]. Basically, to obtain a representative set of compounds that cover a wide chemical space, the Abraham descriptor values (excess molar refraction, dipolarity/polarizability, hydrogen bond acidity and basicity, and McGowan volume) of the compounds were considered [35]. To this end, a total of 2975 compounds of different natures were analyzed according to their descriptor values through a principal component analysis. Then, the 2975 compounds were plotted according to the two main principal component values (which represent the highest variance in the system). This plot provided a map of compounds distributed according to their physicochemical properties. The final selection of 63 compounds was performed, trying to cover all the regions of the plot. Additional requirements were that the selected compounds must have a chromophore group to be compatible with the detection system, and must be neutral at the pH of the determination.

The substances displaying the highest logP values in this table are butylbenzene for both logP_SC_ and logP_HTAB_, and 1-phenylheptan-1-one for logP_LPFOS_. On the other hand, pyrimidine shows the lowest logP value for logP_SC_, 4-aminobenzamide for logP_HTAB_, and hydroquinone for logP_LPFOS_. The logP_SC_ and logP_LPFOS_ exhibit more similar values compared to logP_HTAB_, indicating a possible correlation between logP_SC_ and logP_LPFOS_. Furthermore, a general trend is noticed: compounds with higher hydrophobicity tend to have higher logP values, while those with lower values are more hydrophilic. Therefore, the logP values for the three types of micelles serve as measures of the lipophilicity or hydrophobicity of the respective compounds.

### 2.2. Correlation of logP Values in Micelles Using DFT Calculations

**Figure 1:**
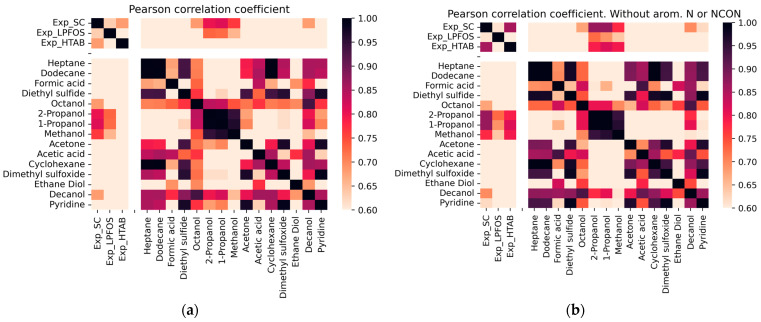
Heatmap of pairwise correlation of logP values for experimental and B3LYP calculated predictions. Three first logP values are the experimental values in SC, LPFOS and HTAB micelles, respectively. The heat map is colored by the significance of Pearson coefficient, where a darker red indicates a higher degree of correlation. In (**a**) all compounds are used, in (**b**) molecules with Nitrogen in an aromatic ring or with the urea (carbamide) group are excluded.

Figure 1 shows the correlation coefficient values among the experimental and calculated logP in 15 different solvent–water combinations, where a darker color represents a higher correlation coefficient. Molecular representation of all solvents used in DFT calculations are shown in Table S1. It can be seen in Figure 1a that the experimental logPs in SC and HTAB show high correlation between them and with some calculated logP_solv/water_ values. However, the logP in HTAB is not correlated with any combination of computed logP values. With respect to the experimental logPs in SC and LPFOS, the highest correlation of computed logP is obtained with propan-1-ol or propan-2-ol solvents. It seems that a curious pattern can be observed for the calculated logP_octanol/water_. While it is highly correlated with the experimental logP_SC_ values, it also exhibits a high correlation with all other calculated logP values for different solvent combinations.

In Figure 1b, a new heatmap is presented that shows the pairwise correlation between experimental and calculated logP values, but with the exclusion of compounds containing nitrogen in an aromatic ring or the urea group. It is observed that all experimental logP values, including logP_HTAB_, show a high correlation with propan-2-ol and propan-1-ol. Additionally, for logP_SC_ and logP_HTAB_, a high correlation with methanol is also observed. This suggests that the excluded compounds may have a different mechanism for describing the partition coefficient of the HTAB micelle.

An analysis is performed comparing calculated and experimental partition coefficients for the HTAB, SC, and LPFOS micelles. The results of the linear regression analysis for the partition coefficients of propan-1-ol/water, propan-2-ol/water, and methanol/water are presented in Table 3. The best correlation is observed for SC micelles. The partition coefficient calculated for propan-1-ol compared to the experimental partition coefficient of SC micelles provided the best correlation (R^2^ of 0.67). It can be seen that the SC and LPFOS micelles behave similarly to aqueous mixtures with alcoholic solvents with dielectric constants ranging from 20 to 33. It needs to be mentioned that because these solvents are miscible with water, the partition coefficient of these solvents cannot be evaluated using the traditional shake flask technique. Alternatively, these coefficients can be determined through the application of appropriate thermodynamic cycles and using immiscible solvents. With respect to HTAB micelles, the prediction is improved for compounds that do not contain nitrogen in an aromatic ring or the urea group.

Table 3 presents a predictive tool that facilitates the identification of the most suitable micellar system for carrying a specific drug. By employing the equations provided in this table, it becomes possible to make a comparison of the LogP values among the three types of micelles (HTAB, SC, and LPFOS). This comparison enables the determination of which micellar system would yield a higher LogP value for the particular drug being considered. Finally, this predictive approach helps in selecting the most appropriate micelle for drug delivery and optimizing drug formulation and efficacy.

### 2.3. Estimation of logP Values in Micelles Using SVM Calculations

A k-means clustering was performed on the set of compounds using a collection of 85 chemical descriptors to analyze the data (Table S3). In the present study, the determined number of partitioning patterns (clusters) was three. It can be concluded that the partitioning into three categories is related to specific features characteristic of each obtained class.

**Figure 2:**
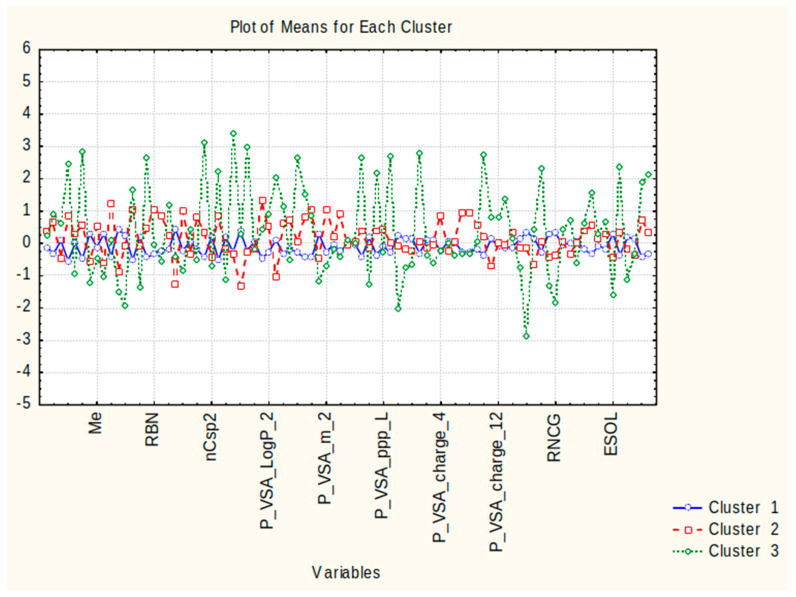
Plot of means for each variable for each identified cluster. Due to lack of space, only 10 of the descriptor names are plotted but the order is the same as in the input matrix (of variables); the distance between the plotted variables is 8 spaces.

Cluster 1 contains 45 out of all 63 compounds (approximately 70% of the cases). The members do not differ substantially with respect to their structural and molecular descriptors, whose values are on a medium level (see Figure 2) without an expressed minima or maxima of their absolute (standardized) values. It may be assumed that this pattern of objects is a specific “medium” with respect to the descriptor values, is characterized by a good consistency of indication, and could be called, conditionally, a “mixed compound” pattern.

Cluster 2 consists of 13 members out of a total of 63 cases, accounting for approximately 20% of all cases. It is important to emphasize that this cluster predominantly comprises representative pesticide compounds and can be conditionally referred to as the ”pesticide compounds” pattern. The pattern exhibits specific feature characteristics (Figure 2) that are responsible for its partitioning. These characteristics include maximal levels for the descriptors GD, RBN, H%, PVS_A_m2, P_VSA_e2, P_VSA_i2, P_VSA_charge3, P_VSA_charge8, and P_VSA_charge9, as well as minimal levels for the descriptors N%, MCD, and P_VSA_LogP_3.

Cluster 3 consists of only 5 members out of a total of 63 cases, accounting for approximately 7%. All the members belong to hormone compounds, and the conditional name for this cluster should be the “hormonal compounds” pattern. It is characterized by significantly different values of the descriptors compared to those of clusters 1 and 2. Twenty-six of the descriptors indicate maximal values, while the other twenty-six indicate minimal values. This represents a typical case of an “outlying cluster”, further supporting the conclusion that this group of objects is markedly different from the rest.

Figure 2 displays the averages for each descriptor of the three identified clusters, effectively demonstrating the differences between them and the descriptors responsible for this partitioning.

**Figure 3:**
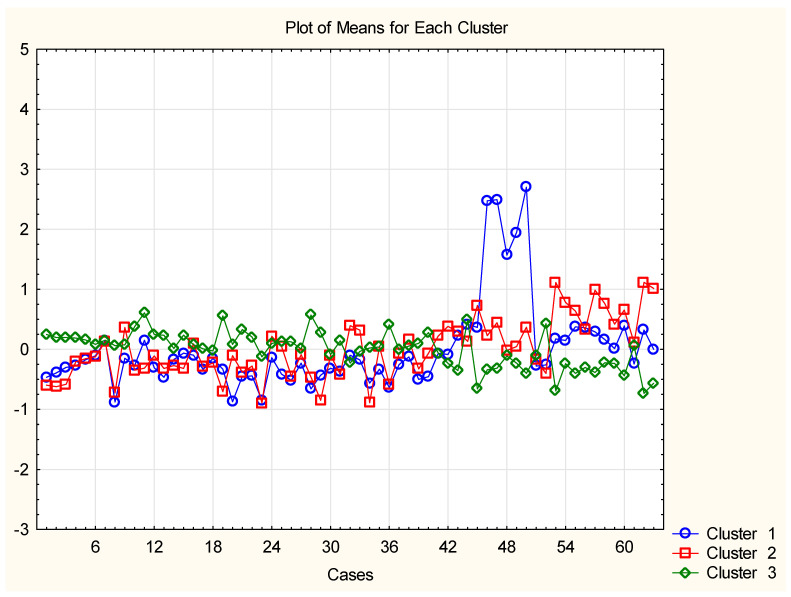
Plot of means (after standardization of the input matrix) of each of the cases for each of the identified patterns of variables; latent factor 1 is marked with blue color, latent factor 2—with red color, latent factor 3—with green color. The plot gives an idea of the relationships between cases and variables.

Furthermore, a PCA was conducted to explore the partitioning among the 85 descriptors. PCA is a widely used chemometric technique that involves projecting the original variables onto new, orthogonal directions known as latent factors. These factors are linear combinations of the original variables, and their associated factor loadings determine their impact on the analysis. The resulting factor scores represent the new coordinates of the objects in the reduced-dimensional space. The PCA analysis revealed that three latent factors accounted for more than 70% of the total variance, as shown in Table S3 (factor loadings table). Figure 3 shows that the most significant set of cases is explained by the highest loadings in factor 1 (all three experimental parameters are included in one factor). The second factor consists of the highest loadings for the second big set of objects, and the outlying set of objects is related to the highest loadings in factor 3.

This work uses supervised and unsupervised machine learning methods to predict the logP values for different micelle formations. SVM calculations were applied as a regression method. The obtained results are presented in Table 4. The regression model was developed based on the list of descriptors presented in Table 4. Grid searches were performed using 10-fold cross-validation. The main descriptors were obtained from the class of molecular descriptors known as P_VSA descriptors, which quantify the van der Waals surface area (VSA) with a specific property P within a certain range [36]. For model development, 85% of the data was used for training, while the remaining 15% was reserved for testing. It is evident that the selected SVM model and the list of desired features yield a significantly high prediction rate for logP.

Our study demonstrates that SVM is a powerful machine learning model capable of predicting logP values from both high-dimensional and low-dimensional data spaces based on the selective nature of the descriptors.

## 3. Materials and Methods

### 3.1. Regents and Materials

Phosphoric acid (85% in water), lithium hydroxide (98%), sodium dihydrogen phosphate monohydrate (G.R.), disodium hydrogen phosphate (G.R.), sodium hydroxide (G.R.), phenyl-undecyl ketone (98%), and methanol (for chromatography) were obtained from Merck. SC (>98%), HTAB (>99%), and LPFOS (25% in water) were obtained from Fluka. Water was Milli-Q plus (Millipore) with resistivity of 18.2 MΩ cm. The test solutes were reagent-grade or better and obtained from several makers.

### 3.2. Determination of Partition Coefficients in Systems of SC, LPFOS, and HTAB Micelles

MEKC analyses were conducted using a UV diode array detector in a Beckman P/ACE System 5500 capillary electrophoresis instrument, with a fused silica capillary of 47 cm total length (40 cm effective length) and 50 μm internal diameter. The measurements were carried out at 25 °C and +15 kV for the anionic surfactants (SC and LPFOS) and −15 kV for the cationic one (HTAB). Detection was set at 214 nm. To inject the test compounds into the capillary, a pressure of 0.5 p.s.i. was applied for 1 s.

The capillary was prepared through a conditioning process, which involved flushing with water for 5 min, treating with 1 M sodium hydroxide solution for 20 min, followed by a rinse with water for 10 min, treatment with 0.1 M sodium hydroxide solution for 10 min, and, finally, treatment with separation buffer for 20 min. Before each injection, the capillary was rinsed with a separation buffer for 5 min.

Three different micelle solutions were prepared at pH 7: 80 mM of SC, 40 mM of LPFOS, and 20 mM of HTAB, all three in 20 mM phosphate buffer. Test compounds were dissolved in a methanol solution (used as an electro-osmotic flow marker), which already contained 2 mg mL^−1^ of phenyl-undecyl ketone (used as a micellar marker). The concentration of the test compounds was 2 mg mL^−1^. All solutions were filtered through 0.45 μm nylon syringe filters (Albet). All measurements were performed in triplicate.

In MEKC, the separation of neutral molecules occurs based on their partitioning between the micellar phase and the aqueous phase. The retention factor (*k*) of a compound can be determined using the following formula:

(1) $$k=\frac{t_{R}-t_{0}}{t_{0}\left(1-t_{R}/t_{m}\right)}$$

The retention time (*t_R_*) for the specific compound being analyzed is measured, while the retention times of the electro-osmotic flow and micellar markers (methanol and phenyl-undecyl ketone, respectively) are denoted as *t*_0_ and *t_m_*.

In this particular study, partition coefficients between water and SC, LPFOS, and HTAB micelles were determined by utilizing previously obtained retention times [13] and applying the following formula:

(2) $$k=\frac{\upsilon \left(C_{T}-CMC\right)}{1-\upsilon \left(C_{T}-CMC\right)}$$

where *P* is the partition coefficient, and *C_T_* is the total surfactant concentration (80 mM for SC, 40 mM for LPFOS, and 20 mM for HTAB). The *CMC* values in 20 mM phosphate buffer (pH 7) were experimentally determined in previous works and are 12.4 mM for SC, 3.27 mM for LFPOS, and 0.34 mM for HTAB [37]. U is the partial molar volume of the surfactants and has a value of 0.317 L mol^−1^ for SC [38], 0.285 L mol^−1^ for LPFOS [39], and 0.324 L mol^−1^ for HTAB [40].

### 3.3. QM Computational Determination of Partition Coefficients

The computations in this study were conducted using the Gaussian 16 (Revision C.01) [41] quantum chemistry software package to calculate solvation free energies and various molecular properties. The Avogadro cross-platform molecule editor was utilized to generate all molecular structures, and only the more extended conformation was used for each compound. Specifically, the focus of this work was to determine the solvation free energy of 63 compounds in 16 different solvents.

In this study, the B3LYP calculations were performed with the 6.311++G** basis set to optimize the geometries of all compounds. The solvation model based on SMD was employed to predict the partition coefficient of the molecules in different solvents. This model divides the solvation free energy into two main contributions, bulk electrostatic and cavity dispersion contributions, making it a widely applicable and universal solvation model that can be used for any solute (neutral and charged) in a variety of parametrized solvents.

In order to determine the solvent–water partition coefficient, the compounds were optimized to obtain their minimum energy at a pressure of 1 atm and temperature of 298.15 K, while ensuring that all vibrational frequencies were positive. The calculation of the Gibbs free energy associated with the transfer of solutes between the solvent and water phases is fundamental in determining the partition coefficient. The logarithm of the partition coefficient (logP) is directly proportional to the difference in solvation free energies (∆*G*°*_solv/wat_*):

(3) $${\Delta G{}^{\circ}}_{\frac{solv}{wat}}={\Delta G{}^{\circ}}_{solv}-{\Delta G{}^{\circ}}_{wat}$$

(4) $$\mathrm{logP}=\frac{{-\Delta G{}^{\circ}}_{\frac{solv}{wat}}}{RTln\left(10\right)}$$

where *R* is the molar gas constant and *T* is the temperature (298 K). We employed the same procedure for SC, LPFOS, and HTAB that we applied for SDS micelles [42].

### 3.4. Correlation Analysis

Heatmaps of Pearson correlation coefficients were produced to identify correlation between variables. Each variable is represented by a colored square, with the color indicating the strength and direction of the correlation between that variable and every other variable in the dataset. The Pearson correlation coefficient ranges from −1 to +1, with −1 indicating a perfect negative correlation, 0 indicating no correlation, and +1 indicating a perfect positive correlation. The heatmap allows us to quickly identify patterns and relationships between variables, as highly correlated variables will appear as blocks of similar color in the heatmap.

### 3.5. Supervised and Unsupervised Methods

Linear regression analysis was conducted using python tools to calculate coefficients, confidence intervals, standard errors, F statistics, significant data of partition coefficient and F, as well as Pearson’s correlation coefficient. Additionally, the accuracy of the regression model’s prediction of experimental octanol/water logP values was evaluated through the computation of statistical measures, including the mean absolute error (MAE), mean square error (MSE), and root mean square error (RMSE).

### 3.6. K-Means Clustering

K-means clustering is a well-documented supervised machine learning pattern recognition procedure [43,44]. It requires an a priori determined number of clusters to which the objects of interest should be partitioned. The hypothesis for the predetermined number of clusters follows expert opinion or specific reasons of the researcher (preliminary information, preliminary testing, etc.). The major goal of this statistical method is to partition the objects of interest into patterns of similarity (clusters) whose number is in line with the preliminary hypothesis. The algorithm used relies on minimization of the within-group distances (usually squared Euclidean distances). Cluster centers (centroids) are used to find groups of comparable special distribution.

The input matrix is of the dimension 63 cases × 85 variables. The raw data were subject to a standardization procedure (z-transform) to avoid differences in variable dimensions. The goal of the partitioning procedure was to reveal patterns of similarity within the 63 compounds of interest and, further, to determine the descriptors contributing mostly to the partitioning. The structure of the dataset was based on the generated descriptors retrieved using the AlvaDesc v.2 software (Milano, Italy) [45].

### 3.7. Principal Component Analysis (PCA)

PCA is a typical projection method based on the reduction of the dimensionality of the system under consideration. This makes it possible to present on a plot the relationship between the variables (using the values of the factor loadings) or between the objects (using the calculated factor scores as the new coordinates of the objects). The reduction of the dimensionality of the initial large dataset enhances the interpretability of the original system while preserving a high amount of explained variance from the original set. The algorithm achieves this through the decomposition of the starting large data matrix into a smaller number of principal components (latent variables), which are linear combinations of the original variables representing directions in space.

## 4. Conclusions

This study aimed to determine a methodology for predicting the experimental partition coefficients (Log P) for a diverse set of compounds for three different micelle formulations: SC, LPFOS, and HTAB. The obtained LogP values were used to parametrize computational methodologies for each type of micelle. Correlations between experimental and calculated logP values were examined using simple DFT calculations, and the SVM regression model was built based on relevant descriptor space. This predictive approach could have significant implications for drug delivery and formulation optimization. It can be used to identify the micellar system that offers a higher Log P value, thus enhancing the potential for successful therapeutic applications.

When considering the entire set of compounds, the results revealed an increased correlation between experimental logP values and the DFT predictions for the SC and LPFOS micelle systems obtained for the propan-1-ol/water or propan-2-ol/water solvent mixtures. However, the logP values in HTAB were not correlated with any of the calculated logP values. It has been found that compounds containing nitrogen in an aromatic ring, or the urea group, exhibited a different mechanism in describing the partition coefficient of the HTAB micelle. Excluding these compounds from the set, the best correlation was observed for all micelles in the propan-1-ol/water or propan-2-ol/water solvent mixtures.

Furthermore, SVM calculations and k-means clustering were conducted using a set of 85 descriptors. The findings imply that the partitioning into three classes is coupled to specific features that are characteristic of each obtained class. These results provide valuable insights into the behavior of different types of micelles and can contribute to the development of more accurate computational methods for predicting partition coefficients in micelles.

## Figures and Tables

**Table 1:** Chemical structure of the molecules that form the micelles of this study.

Micelle Name	Symbol	Structure	Schematic Representation of Formed Micelles
Hexadecyltrimethyl- ammonium bromide	HTAB	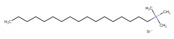	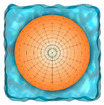
Lithium perfluorooctanesulfonate	LPFOS	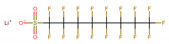	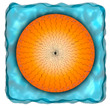
Sodium cholate	SC	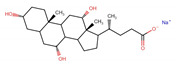	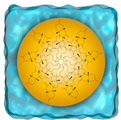

**Table 2:** List of experimental partition coefficients of compounds in SC, LPFOS, and HTAB micelles (LogP_SC_, LogP_LPFOS_, LogP_HTAB_) determined from retention factors obtained from MECK experiments with 80 mM of SC, 40 mM of LPFOS, and 20 mM of HTAB, all in 20 mM phosphate buffer at pH 7 at 25 °C.

Compound	logP_SC_	logP_HTAB_	logP_LPFOS_
Ethylbenzene	2.50	3.00	2.06
Propylbenzene	2.94	3.42	2.39
Butylbenzene	3.26	3.71	2.71
1-Phenylethanone	1.33	2.03	2.19
1-Phenylpropan-1-one	1.65	2.42	2.44
1-Phenylbutan-1-one	2.01	2.80	2.72
1-Phenylpentan-1-one	2.41	3.24	3.01
1-Phenylheptan-1-one	3.15	-	3.68
Furan	0.77	1.48	1.19
2-Nitroaniline	1.59	2.67	1.80
2,3-Benzofuran	2.12	2.82	1.82
Diphenylmethanone	2.48	3.28	3.01
Benzamide	1.06	1.72	1.50
4-Chloroaniline	1.69	2.69	1.44
2,3-Dimethylphenol	1.90	3.15	1.66
Naphtalen-2-ol	2.31	-	1.73
4-Aminobenzamide	0.98	1.11	1.76
3-Methylphenol	1.53	2.78	1.43
2,4-Dimethylphenol	1.93	3.17	1.02
Naphthalene	2.67	3.47	2.09
Pyrimidine	0.56	-	1.27
Benzaldehyde	1.20	1.91	1.91
3-Chloroaniline	1.63	2.72	1.41
Pyrrole	0.68	1.65	0.72
3-Nitroaniline	1.38	2.42	1.53
4-Chlorophenol	2.00	3.24	1.30
Phenol	1.21	2.35	1.08
Methylbenzoate	1.71	2.39	2.36
Bromobenzene	2.37	2.95	1.80
1,4-Xylene	2.51	3.04	2.10
Benzene-1,3-diol	1.21	2.48	0.75
2-Methylaniline	1.17	2.15	1.59
Aniline	0.92	1.83	1.34
Nitrobenzene	1.47	2.21	1.94
Chlorobenzene	2.21	2.77	1.77
*N*-4-chlorophenylacetamide	2.03	2.80	1.84
*N*-Phenylacetamide	1.25	1.98	1.58
4-Nitroaniline	1.52	2.50	1.45
Anisole	1.66	2.31	1.83
Benzonitrile	1.21	1.96	1.95
1-Ethyl-4-nitrobenzene	2.19	3.02	2.68
Benzyl benzoate	2.99	-	3.18
Caffeine	1.11	1.32	1.85
Corticosterone	1.94	3.69	3.64
Cortisone	1.72	3.16	3.37
β-Estradiol	2.77	-	2.84
Estriol	2.32	3.52	2.01
Cortisol	1.83	3.39	2.89
Hydroquinone	1.09	1.94	0.19
Quinoline	1.65	2.36	2.68
Atrazine	1.86	1.90	2.71
Diuron	2.46	2.19	2.34
Isoproturon	2.19	1.95	2.61
Linuron	2.59	2.24	2.50
Metobromuron	2.16	2.03	2.22
Monuron	1.81	1.73	2.03
Metoxuron	1.69	1.46	2.34
Phenylurea	1.20	1.20	1.38
Propazine	2.02	2.08	3.03
Fluometuron	2.01	1.92	2.57
*N,N*-Diethyl-4-nitroaniline	2.44	3.56	3.36
1-Methoxy-4-nitrobenzene	1.69	2.58	2.20
1-Methoxy-2-nitrobenzene	1.55	2.37	2.26

**Table 3:** Best linear regressions obtained to predict the logP in SC, LPFOS, and HTAB micelles using DFT calculations. Results from B3LYP functional with 6-31++G** basis set using SC, LPFOS, and HTAB for propan-1-ol, propan-2-ol, and methanol are indicated. x refers to predicted logP alcohol/water, and y refers to the predicted logP in micelles. *N set: compounds containing nitrogen in an aromatic ring or the urea group are excluded.

Micelle	Solvent	B3LYP
LPFOS	Propan-1-ol	y = 0.46x + 0.77 R^2^ = 0.52 MAE = 0.87
	Propan-2-ol	y = 0.49x + 0.61 R^2^ = 0.53 MAE = 0.92
	Methanol	y = 0.41x + 0.90 R^2^ = 0.43 MAE = 0.86
SC	Propan-1-ol	y = 0.47x + 0.55 R^2^ = 0.67 MAE = 0.92
	Propan-2-ol	y = 0.46x + 0.51 R^2^ = 0.64 MAE = 1.08
	Methanol	y = 0.41x + 0.68 R^2^ = 0.58 MAE = 0.89
HTAB	Propan-1-ol	y = 0.23x + 1.80 R^2^ = 0.13 MAE = 0.74
	Propan-2-ol	y = 0.22x + 1.83 R^2^ = 0.1 MAE = 0.72
	Methanol	y = 0.24x + 1.78 R^2^ = 0.13 MAE = 0.72
HTAB without N set *	Propan-1-ol	y = 0.56x + 1.24 R^2^ = 0.66 MAE = 0.45
	Propan-2-ol	y = 0.54x + 1.24 R^2^ = 0.62 MAE = 0.43
	Methanol	y = 0.56x + 1.26 R^2^ = 0.63 MAE = 0.46

* Molecules with Nitrogen in an aromatic ring or with the urea (carbamide) group are excluded.

**Table 4:** SVM regression results for LogP prediction in SC, HTAB, and LPFOS micellar systems.

	SC	HTAB	LPFOS
Variables (descriptors)	Mv, RBN, RBF, H%, N%, O%, NRS, nR09, nR10, X4Av, P_VSA_LogP_2, P_VSA_s_4, P_VSA_ppp_P, P_VSA_charge_1, P_VSA_charge_3, P_VSA_charge_4, P_VSA_charge_5, P_VSA_charge_13, P_VSA_charge_14	nSK, nH, N%, Xu, S1K, DELS, BAC, X0, X0sol, P_VSA_LogP_1, P_VSA_LogP_4, P_VSA_LogP_6, P_VSA_LogP_8, P_VSA_MR_5, P_VSA_m_5, P_VSA_s_3, P_VSA_ppp_D, P_VSA_charge_2, P_VSA_charge_4, P_VSA_charge_5, P_VSA_charge_6, P_VSA_charge_9, P_VSA_charge_12, P_VSA_charge_14, qpmax, qnmax, Qpos, Qneg, Qtot, Qmean, Q2, RPCG, RNCG, TPSA(NO), TPSA(Tot)	RBF, nTB, MaxTD, P_VSA_LogP_2, P_VSA_LogP_3, P_VSA_LogP_4, P_VSA_MR_2, P_VSA_s_3, P_VSA_charge_2, P_VSA_charge_6, P_VSA_charge_7, P_VSA_charge_9, P_VSA_charge_13, P_VSA_charge_14, Qmean
R^2^	0.693	0.565	0.783
RMSE	0.369	0.248	0.202
MAE	0.318	0.241	0.304

## Data Availability

The data that support the findings of this study are available on request from the corresponding author.

## Supplementary Materials

The following supporting information can be downloaded at https://www.mdpi.com/article/10.3390/molecules28155729/s1, Table S1: Molecular representation of non-aqueous solvents used in DFT; Table S2: Linear regression parameters obtained for the correlation of the calculated LogP in 15 different solvents with respect to the experimental partition coefficients in SC, LPFOS, and HTAB micelles; Table S3: Factor loadings (Varimax-normalized) of the chemical descriptors obtained from PCA.
