# Supplementary material for: Collection of Partition Coefficients in Hexadecyltrimethylammonium Bromide, Sodium Cholate, and Lithium Perfluorooctanesulfonate Micellar Solutions: Experimental Determination and Computational Predictions
Source: Molecules. 2023 Jul 28;28(15):5729. doi: 10.3390/molecules28155729 (PMC10420229; doi:10.3390/molecules28155729)
Supplement: Supplementary file 1 [file molecules-28-05729-s001.zip › molecules-2522330-supplementary.pdf]

SUPPLEMENTARY INFORMATION

Collection of Partition Coefficients in hexa decyltrimethylammonium bromide (HTAB), sodium cholate (SC), and lithium perfluorooctanesulfonate (LPFOS) micellar solutions. Experimental determination and computational predictions.

Leila Saranjam, Miroslava Nedyalkova, Elisabet Fuguet, Vasil Simeonov, Francesc Mas and Sergio Madurga

Table S1. Molecular representation of non aqueous solvents used in DFT calculations.

**Solvent**	**Structure**
Heptane	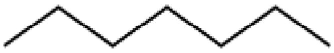
Cyclohexane	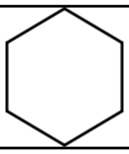
n-Dodecane	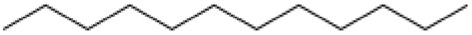
Pyridine	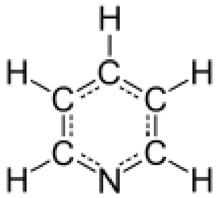
Diethyl sulfide	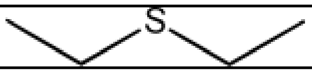
Acetic acid	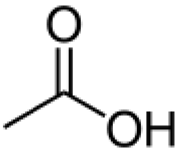
Decan-1-ol	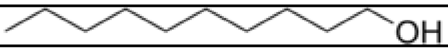
Octanol	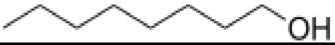
Propan-2-ol	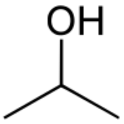
Acetone	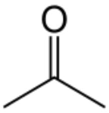
Propan-1-ol	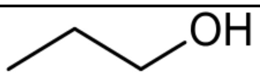
Methanol	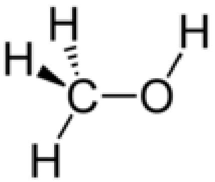
1,2-Ethane diol	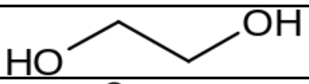
Dimethyl sulfoxide	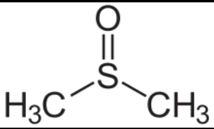
Formic acid	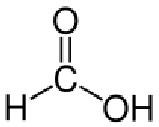

Table S2. Linear regression parameters obtained for the correlation of the calculated LogP in 15 different solvents with respect to the experimental partition coefficients in SC, LPFOS and HTAB micelles. Solvents are sorted as a function of their dielectric constant.

Solvent	Dielectric constant	R2 SC B3LYP	R2 LPFOS B3LYP	R2 HTAB B3LYP
Heptane	1.92	0.002	0.28	0.11
Cyclohexane	2.02	0.3	0.14	0.14
N-dodecane	2.03	0.2	0.15	0.09
Pyridine	2.35	0.3	0.18	0.0034
Diethyl sulfide	6.14	0.26	0.09	0.0019
Acetic acid	6.2	0.16	0.01	0.01
Decan-1-ol	7.53	0.46	0.11	0.06
Octanol	10.3	0.46	0.11	0.09
Propan-2-ol	19.26	0.64	0.53	0.1
Acetone	20.16	0.32	0.17	0.01
Propan-1-ol	21.03	0.67	0.52	0.13
Methanol	32.61	0.58	0.43	0.13
1,2-ethan diol	40.24	0.17	0.0003	0.06
Dimethyl sulfoxide	46	0.22	0.13	0.0008
Formic acid	58	0.17	0.0004	0.015

Table S3. Factor loadings (Varimax normalized) of the chemical descriptors obtained from PCA. Loadings indicated in bold are significant.

Variable	Factor 1	Factor 2	Factor 3
Exp_SC	0.684	0.123	-0.035
Exp_LPFOS	0.627	0.054	-0.228
Exp_HTAB	0.624	0.136	0.336
MW	0.917	0.101	-0.221
AMW	-0.283	0.059	-0.167
Sp	0.971	0.109	-0.068
Mv	-0.532	0.242	0.286
Me	-0.006	-0.232	-0.312
Mp	-0.516	0.372	0.344
Mi	0.309	-0.356	-0.758
GD	-0.8	-0.303	0.298
nAA	-0.522	0.712	-0.062
nTA	0.792	-0.187	-0.336
nBM	-0.296	0.758	0.028
SCBO	0.912	0.206	-0.022
RBN	0.415	0.32	-0.722
RBF	0.237	0.332	-0.705
H%	0.437	-0.059	-0.076
C%	-0.401	0.398	0.757
N%	-0.09	-0.247	-0.762
O%	0.137	-0.235	0.209
X%	-0.029	0.112	-0.335
nCsp3	0.937	-0.164	0
nCsp2	-0.137	0.802	0.408
Fsp3	0.85	-0.11	-0.357
max_conj_path	-0.258	0.703	0.145
nCIR	0.775	-0.126	0.347
MCD	-0.425	-0.057	0.725
RFD	0.754	-0.088	0.374
NRS	0.038	0.763	0.026
P_VSA_LogP_1	0.538	-0.052	-0.593
P_VSA_LogP_2	0.449	0.061	-0.071
P_VSA_LogP_3	0.33	0.162	0.746
P_VSA_LogP_4	0.472	-0.245	-0.262
P_VSA_LogP_5	-0.003	0.101	-0.492
P_VSA_MR_1	0.844	0.162	0.378
P_VSA_MR_2	0.722	-0.207	-0.437
P_VSA_MR_5	0.515	-0.111	-0.715
P_VSA_MR_6	-0.341	0.561	0.4
P_VSA_m_2	0.113	0.376	-0.72
P_VSA_m_5	-0.076	0.123	-0.051
P_VSA_v_3	0.171	0.774	-0.247
P_VSA_e_2	0.223	0.85	0.327
P_VSA_i_2	0.162	0.806	0.265
P_VSA_s_3	0.937	0.142	0.062
P_VSA_s_4	-0.219	0.813	0.276
P_VSA_s_6	0.689	-0.357	-0.121
P_VSA_ppp_L	0.1	0.701	-0.149
P_VSA_ppp_A	0.761	-0.348	0.146
P_VSA_ppp_ar	-0.58	0.758	-0.166

P_VSA_ppp_con	-0.121	0.744	0.001
P_VSA_ppp_cyc	-0.249	0.77	-0.034
P_VSA_charge_1	0.833	-0.283	0.183
P_VSA_charge_2	0.002	0.382	0.05
P_VSA_charge_3	-0.167	0.136	0.033
P_VSA_charge_4	0.018	0.12	-0.558
P_VSA_charge_5	-0.086	0.086	0.073
P_VSA_charge_6	-0.13	-0.022	0.005
P_VSA_charge_7	0.055	-0.064	-0.758
P_VSA_charge_8	0.082	-0.102	-0.863
P_VSA_charge_9	0.236	0.24	-0.112
P_VSA_charge_10	0.856	-0.085	0.298
P_VSA_charge_11	0.115	0.542	0.586
P_VSA_charge_12	0.283	-0.162	-0.106
P_VSA_charge_13	0.492	-0.086	0.236
P_VSA_charge_14	0.321	0.373	0.219
qnmax	-0.452	-0.253	-0.333
Qneg	-0.929	-0.102	-0.311
Qmean	0.129	0.229	0.711
Q2	0.792	0.177	0.406
RPCG	-0.421	0.167	0.307
RNCG	-0.651	-0.221	0.048
SPP	0.321	0.358	0.391
LDI	0.284	0.166	0.503
Ui	-0.008	0.613	0.088
Hy	0.19	-0.444	-0.484
TPSA(NO)	0.573	-0.389	-0.389
ALOGP	0.212	0.63	0.048
LOGP99	0.37	0.66	0.12
ESOL	-0.59	-0.577	-0.041
SAacc	0.741	-0.386	-0.114
PDI	-0.397	0.553	0.076
BLTF96	-0.282	-0.787	0.14
SAscore	0.778	-0.14	-0.392
PBF	0.77	0.234	-0.031
Expl.Var %	34.4	25.4	15.5
